# Association of Primary Care Characteristics with Variations in Mortality Rates in England: An Observational Study

**DOI:** 10.1371/journal.pone.0047800

**Published:** 2012-10-24

**Authors:** Louis S. Levene, John Bankart, Kamlesh Khunti, Richard Baker

**Affiliations:** 1 East Leicester Medical Practice, Leicester, United Kingdom; 2 National Institute for Health Research Collaboration for Leadership in Applied Research and Care for Leicestershire, Northamptonshire and Rutland, Department of Health Sciences, University of Leicester, Leicester, United Kingdom; The University of Texas M. D. Anderson Cancer Center, United States of America

## Abstract

**Background:**

Wide variations in mortality rates persist between different areas in England, despite an overall steady decline. To evaluate a conceptual model that might explain how population and service characteristics influence population mortality variations, an overall null hypothesis was tested: variations in primary healthcare service do not predict variations in mortality at population level, after adjusting for population characteristics.

**Methodology/Principal Findings:**

In an observational study of all 152 English primary care trusts (geographical groupings of population and primary care services, total population 52 million), routinely available published data from 2008 and 2009 were modelled using negative binomial regression. Counts for all-cause, coronary heart disease, all cancers, stroke, and chronic obstructive pulmonary disease mortality were analyzed using explanatory variables of relevant population and service-related characteristics, including an age-correction factor. The main predictors of mortality variations were population characteristics, especially age and socio-economic deprivation. For the service characteristics, a 1% increase in the percentage of patients on a primary care hypertension register was associated with decreases in coronary heart disease mortality of 3% (95% CI 1–4%, p = 0.006) and in stroke mortality of 6% (CI 3–9%, p<0.0001); a 1% increase in the percentage of patients recalling being better able to see their preferred doctor was associated with decreases in chronic obstructive pulmonary disease mortality of 0.7% (CI 0.2–2.0%, p = 0.02) and in all cancer mortality of 0.3% (CI 0.1–0.5%, p = 0.009) (continuity of care). The study found no evidence of an association at primary care trust population level between variations in achievement of pay for performance and mortality.

**Conclusions/Significance:**

Some primary healthcare service characteristics were also associated with variations in mortality at population level, supporting the conceptual model. Health care system reforms should strengthen these characteristics by delivering cost-effective evidence-based interventions to whole populations, and fostering sustained patient-provider partnerships.

## Introduction

Population mortality rates vary within and between countries with developed health care systems [1]. In England the directly age-standardised rates for all-cause mortality have declined steadily from 790 per 100,000 European Standard population in 1993 to 547 per 100,000 in 2009 [2]. Healthcare in England is free at the point of access [3], and virtually the entire population is registered with a primary care provider; nonetheless, wide variations in mortality rates persist between different areas (from 354 to 766 deaths per 100,000 in 2009 among the then 152 primary care trusts, geographical groupings of population and primary care services, in England) [2].

Characteristics of local populations are important determinants of population mortality. There are associations, at individual and population levels, between increased rates of various mortalities and levels of socio-economic deprivation [4], smoking [5], [6], obesity [7], hypertension [8], and diabetes [9]. Different interventions have a variable effect on some of these characteristics, for example, smoking cessation [10], lowering blood pressure [11], lowering low density lipoprotein cholesterol [12], and low dose aspirin [13], which can reduce the numbers of adverse events, in particular cardiovascular events, in high risk patients.

**Figure 1:**
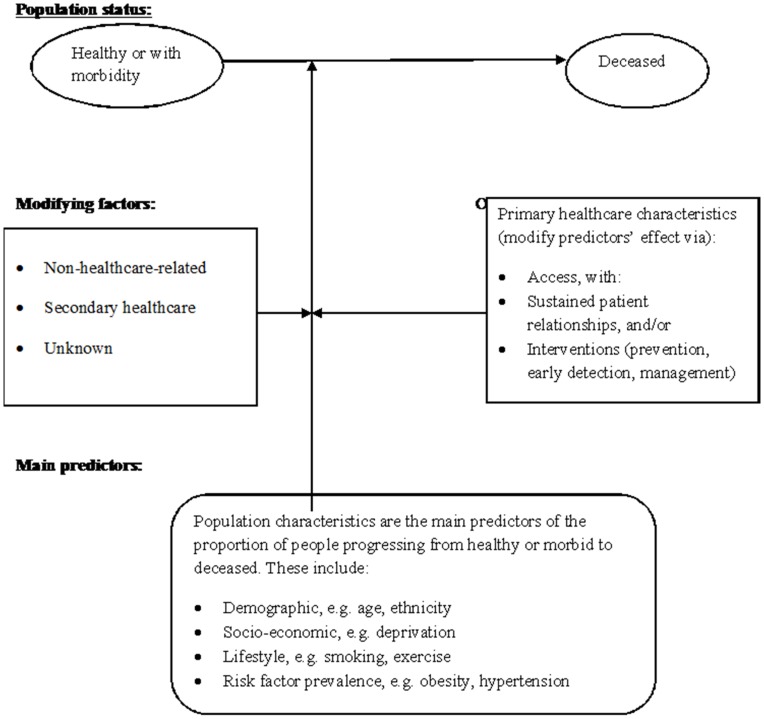
Conceptual model for healthcare and mortality. A proportion of the healthy or morbid population will die each year. This may be predicted by relevant population characteristics; however, appropriate health care may alter this predictive effect, either directly on the progression from 1 or more of these diseases to death or indirectly by affecting a “modifiable” population factor (e.g. detecting and treating blood pressure, detecting obesity, delivering smoking cessation or weight reduction care). In addition to primary healthcare, other factors may affect the progression to mortality, including secondary healthcare and non-healthcare led interventions, such as in education, employment and housing.

Primary care has the potential to improve the effectiveness of health systems by delivering to the majority of the population appropriate preventive measures, and to most of those with common chronic conditions, appropriate management. Primary care has been defined as “the provision of integrated, accessible health care services by clinicians who are accountable for addressing the large majority of personal health care needs, developing a sustained partnership with patients and practising in the context of family and community” [14]. Reviewing the evidence on the contribution of primary care to health systems, Starfield et al [15] identified mechanisms potentially accounting for the beneficial impact of primary care on population health, including greater access to needed services, better quality of care, greater focus on prevention, earlier disease management, and the cumulative effect, with a holistic focus, of greater continuity and comprehensiveness. The rationale for extending access to healthcare in systems where access is not universal is supported by evidence that better access to [16], [17], and greater sustained continuity [18], [19] of healthcare are associated with better health outcomes, especially in long term conditions.

**Table 1:** Annual mortality rates and counts in primary care trusts.

Mortality groupvariable and year	Mean mortalityrates per 1,000population	Minimum mortalityrates per 1,000population	Maximum mortalityrates per 1,000population	95% confidenceintervals for meanper 1,000 population(lower, higher)	Counts Median(Q1, Q3)
All Cause 2009	8.718	4.278	13.160	(8.421, 9.014)	2402 (1640, 3701)
All Cause 2008	9.107	4.161	13.471	(8.804, 9.410)	2498 (1744, 3835)
Coronary Heart Disease 2009	1.288	0.521	2.146	(1.240, 1.336)	355 (241, 550)
Coronary Heart Disease 2008	1.385	0.629	2.253	(1.336, 1.435)	384 (270, 576)
Stroke 2009	0.763	0.261	1.429	(0.728, 0.798)	202 (140, 333)
Stroke 2008	0.815	0.268	1.351	(0.779, 0.851)	215 (148, 353)
Cancer 2009	2.466	1.323	3.899	(2.382, 2.550)	673 (450, 1002)
Cancer 2008	2.485	1.188	3.704	(2.399, 2.571)	668 (466, 1020)
Chronic Obstructive PulmonaryDisease 2009	0.429	0.132	0.750	(0.409, 0.449)	120 (82, 181)
Chronic Obstructive PulmonaryDisease 2008	0.467	0.164	0.822	(0.445, 0.489)	132 (89, 194)

Data available for all 152 primary care trusts in England. These rates are per 1000 population and are not age-standardized (please see text in Methods section). The counts do not have a normal distribution.

At a time of financial constraint, countries are considering how the contribution of primary care to health system efficiency could be improved, for example through improved disease management and prevention that reduces demand for specialist services [3], [20]. The most important recent developments in primary care in England include the introduction of a pay for performance scheme, the Quality and Outcomes Framework, implemented in 2004 with financial incentives to improve quality of care and reduce variations in management of chronic diseases [21], [22], and reforms now underway in which primary care trusts are being replaced by a larger number of general practitioner-led clinical commissioning groups [23]. To improve overall health system performance, evidence on which features of primary care most influence population health, including mortality, should be sought.

Drawing on Starfield’s mechanisms and our previous finding of an association between the lower detection of hypertension by general practices and higher coronary heart disease mortality at primary care trust population level [24], a conceptual model was devised to explain how the effect of population characteristics upon variations in mortality of long-term conditions might be modified by variations in the delivery of primary care that incorporates whole population coverage (greater access to services) and offers sustained relationships with patients (the cumulative effect of primary care delivery characteristics, including continuity and comprehensiveness). Appropriate interventions target both healthy and morbid populations via early detection, prevention and appropriate management of people with established disease. The model also recognises the modifying effect of other factors on mortality, either within secondary healthcare or outside healthcare (e.g. education, housing and employment^4^) (Figure 1). Whilst variations in population mortality are predicted mainly by population characteristics, the model asserts that variations in the delivery of primary care do have some predictive effect, particularly when whole populations are involved.

In order to evaluate this conceptual model, the following testable overall null hypothesis was derived: variations in primary health care do not predict variations in mortality at population level, after adjusting for population characteristics.

## Methods

### Study Design

An observational study was undertaken, involving all primary care trusts in England, in which population and service characteristics were used to explain mortality in 5 disease areas (models) in a two year period using negative binomial regression modelling. Data from the two years were modelled jointly using a generalized estimating equations approach [25], but each model featured only a single outcome, although this outcome was measured twice (once in each year) in the models. The STROBE checklist was used in the design and reporting of this study [26]. We chose to undertake the study at the population level of primary care trusts rather than general practices (of which in England there were 8,305 in the 2009/2010 financial year [27] and 8,229 in 2008/2009 [28]), since we were unable to obtain mortality data at practice level from the Office for National Statistics and reliable data for several population characteristics, such as rates of obesity, smoking and ethnicity, were not available at practice level.

**Table 2:** Characteristics of the explanatory variables.

Variable	2008	2009
*Normally distributed population characteristics*	*Mean (SD)*	*Mean (SD)*
Deprivation indices 2007	23.7 (9.1)	23.7 (9.1)
% of GP list on diabetes register	5 (0.7)	5 (0.8)
*Non-normally distributed population characteristics*	*Median (Q1, Q3)*	*Median (Q1, Q3)*
% White ethnicity	87 (74, 93)	87 (74, 93)
% of adults who were smokers in 2006–8	22 (19, 27)	22 (19, 27)
% of adults who were obese in 2006–8	24 (22, 26)	24 (22, 26)
% of population aged 65 or more years	16 (13, 19)	16 (13, 18)
% of population who are male	49 (48, 50)	49 (48, 50)
*Normally distributed health care characteristics*	*Mean (SD)*	*Mean (SD)*
% of GP registered list on hypertension register	13 (2)	13 (2)
% Patients with recalled perception of being able to seepreferred GP	62 (5)	62 (4)
% Response to GP Patient Survey	39 (7)	40 (7)
% of over 65 s given influenza immunisation	74 (2)	72 (2)
% of NHS smoking cessation clinic attenders self-reportingstopped at 4 weeks	50 (8)	50 (8)
% of CHD patients on aspirin	94 (0.8)	94 (0.9)
% of stroke patients on aspirin	94 (0·7)	94 (0.8)
% of CHD patients with last cholesterol <5 mmol/L	82 (2)	82 (2)
% of stroke patients with last cholesterol <5 mmol/L	77 (3)	77 (2)
% of COPD patients given influenza immunisation	92 (1)	93 (1)

152 Primary Care Trusts in England.

The main sources of data for this study were the Office for National Statistics (for mortality and population data) and the NHS Information Centre for Health and Social Care (for service data), unless otherwise stated.

### Study Sample

The study included all 152 primary care trusts in England into which the country was divided between the years 2006 and 2010. Primary health care was delivered by general practices contracted to the trusts. In 2008, the estimated resident population in England was 51.5 million (the number of people registered with primary care trusts ranging from 90,800 to 1,283,600). In 2009 the estimated resident population in England was 51·8 million (range 90,900 to 1,289,400) [29].

**Table 3:** Negative binomial regression results for mortality groups in 2008–2009.

Explanatory variable	All-cause mortalityIRR (95% CI)P value	All cancersmortalityIRR (95% CI)P value	Coronary HeartDisease mortalityIRR (95% CI)P value	Stroke mortalityIRR (95% CI)P value	Chronic ObstructivePulmonary Diseasemortality IRR(95% CI) P value
Year of mortality counts	1.07 (1.05, 1.09) <0.0001	1.02 (1.0, 1.03) 0.001	1.12 (1.1, 1.2) <0.0001	1.1 (1.07, 1.12) <0.0001	1.07 (1.04, 1.1) <0.0001
Deprivation indices 2007	**1.005 (1.002,1.007)** **<0.0001**	**1.006 (1.004, 1.008) <0.0001**	**1.004** **(1.001, 1.007)** **0.009**	**1.006 (1.001, 1.01) 0.008**	**1.014 (1.01, 1.02) <0.0001**
White ethnicity	**1.007 (1.005, 1.009) <0.0001**	**1.008** **(1.006, 1.009)** **<0.0001**	**1.01 (1.008, 1.02) <0.0001**	**1.009 (1.004, 1.02) <0.0001**	**1.012 (1.01, 1.02) <0.0001**
% of adults who were smokers in 2006–8	1.003 (0.99, 1.01) 0.25	1.001 (0.997, 1.01) 0.55	1.004 (0.99, 1.01) 0.15	0.99 (0.98, 1.01) 0.07	1.008 (1.001, 1.02) 0.06
% of adults who were obese in 2006–8	1.004 (0.99, 1.01) 0·13	**1.005** **(1.001, 1.009)** **0.004**	0.998 (0.99, 1·01) 0.53	**1.01 (1.001, 1.02)** **0.03**	1.008 (0.99, 1.02) 0.·08
% of population who are male	0.99 (0.96, 1.01) 0.16	0.99 (0.97, 1.01) 0.12	0.98 (0.94, 1.01) 0.10	1.02 (0.98, 1.07) 0.21	**0.94 (0.90, 0.99) 0.004**
% of population aged 65 or more years	**1.04 (1.03, 1.05) <0.0001**	**1·04** **(1·03, 1·05)** **<0·0001**	**1.03 (1.02, 1.05) <0.0001**	**1.08 (1.06, 1.10) <0.0001**	**1.01 (1.003, 1.03) 0.01**
% of GP registered list on diabetes register	**1.04 (1.01, 1.07) 0.0004**	1.01 (0.99, 1.03) 0.19	**1.14 (1.09, 1.19) <0.0001**	**1.08 (1.03, 1.13) 0.0001**	Not used
% of GP registered list on hypertension register	0.99 (0.97, 1.01) 0.14	Not used	**0.97** **(0.95,0.99)** **0.006**	**0.94 (0.91, 0.97) <0.0001**	Not used
% of over 65 s given influenza immunisation	0.997 (0.994, 1.01)0.18	Not used	Not used	Not used	Not used
% of NHS smoking cessation clinic attenders self-reporting stopped at 4 weeks	0.999 (0.998, 1.01)0.30	0.999 (0.995, 0.999) 0.19	Not used	0.999 (0.996, 1.001)0.19	0.998 (0.995, 1.01) 0.17
% of patients with recalled perception of being able to see preferred GP	0.999 (0.·997, 1.01)0.92	**0.997** **(0.995, 0.999)** **0.009**	0.999 (0.995, 1.01) 0.77	1.0002 (0.99, 1.01) 0.93	**0.993 (0.98, 0.998) 0.02**
% of CHD patients on aspirin	Not used	Not used	0.99 (0.97, 1.01) 0.22	Not used	Not used
% of stroke patients on aspirin	Not used	Not used	Not used	0.99 (0.96, 1.02) 0.38	Not used
% of CHD patients with last cholesterol <5 mmol/L	Not used	Not used	0.996 (0.98, 1.01) 0.32	Not used	Not used
% of stroke patients with last cholesterol <5 mmol/L	Not used	Not used	Not used	0.999 (0.992, 1.01) 0.99	Not used
% of COPD patients giveninfluenza immunisation	Not used	Not used	Not used	Not used	0.993 (0.98, 1.01) 0.36

Significant predictors in **bold**.

Columns  =  mortality groups, Rows  =  explanatory variables.

In each cell, where there are figures:

In order, the first figures are incident rate ratios (IRR); followed by 95% confidence intervals in parentheses; followed by significance levels.

Statistical model: negative binomial regression, using the log of the primary care trust size as an offset.

GP = general practitioner.

### Variable Selection

#### Mortality outcomes

Mortality counts in each trust were the dependent variables. To maintain variable consistency, counts were used in preference to age-standardized rates, as none of the selected explanatory variables were age-standardized [30]. To correct for variations in trust population age structures, the percentage of a primary care trust’s population aged 65 years or more was one of the explanatory variables.

Mortality counts for all cause (total), coronary heart disease (International Classification of Diseases ICD-10 I20–I25), all cancers (International Classification of Diseases ICD-10 C00–C97), stroke (International Classification of Diseases ICD-10 I60–I69) and chronic obstructive pulmonary disease (International Classification of Diseases ICD-10 J40–J44) were obtained for the last two available years, 2008 and 2009. Coronary heart disease, all cancer, stroke and chronic obstructive pulmonary disease mortality accounted for 14.7%, 28.1%, 8.9% and 4.8% (when combined, more than half) of total mortality in England in 2009, respectively [2]. These mortality groups were selected as, in addition to all-cause mortality, they constituted common long-term conditions potentially amenable to interventions in primary care, including prevention, early detection, and effective management; and data were available for both the mortality group and principal risk factors. Our previous study that contributed to the development of the conceptual model was focused on coronary heart disease [24], but we included coronary heart disease in the current analysis, because different outcomes (counts instead of age-adjusted rates) and some different predictor variables were used.

#### Predictor selection

Predictor selection was governed by two principles: conceptual relevance and the number of observational units in our dataset. Candidate explanatory variables relating to population or primary healthcare service characteristics were initially selected on the basis of the conceptual model (Figure 1), using data available for every trust with potential to predict mortality. However, in order for a regression analysis to be reliable, a maximum of no more than m/10 explanatory variables per analysis is recommended [31], where m is the number of observational units (such as patients). This required selectivity as to which variables were used for modelling each mortality group.

**Table 4:** Effect on variations in mortality of a unit increase in the value of predictors.

Explanatory variable (unit)	All causemortality	All cancersmortality	Coronary Heart Diseasemortality	Strokemortality	Chronic ObstructivePulmonaryDisease mortality
Deprivation indices 2007 (1 unit on scale)	+0.5%	+0.6%	+0.4%	+0.6%	+1.4%
White ethnicity (1%)	+0.7%	+0.8%	+1.0%	+0.9%	+1.2%
%of adults who were obese in 2006–8 (1%)	NS	+0.5%	NS	+1.0%	NS
% of population who are male (1%)	NS	NS	NS	NS	−6.0%
% of population aged 65 or more years (1%)	+4.0%	+4.0%	+3.0%	+8.0%	+1.0%
% of GP registered list on diabetes register (1%)	+4.0%	NS	+14.0%	+8.0%	Not used
% of GP registered list onhypertension register (1%)	NS	Not used	−3.0%	−6.0%	Not used
% patients with recalled perceptionof being able to see preferred GP (1%)	NS	−0.3%	NS	NS	−0.7%

NS =  not significant.

For every 1 unit increase in the predictor, the predicted count changes by (the coefficient minus 1) times 100%.

So, for % of GP registered list on hypertension register in coronary heart disease mortality, for every 1% increase in the register, the predicted count changes by (0.97−1)×100 = −0.03×100 = −3% (a decrease of 3%), and for % of patients with recalled perception of being able to see preferred GP in cancer mortality, for every 1% increase in being able to see preferred GP, the predicted count decreases by (0.997−1)×100 = −0.3% (a decrease of 0.3%).

#### Population predictors

Population data at primary care trust level were obtained at mid-year in 2008 and 2009 for numbers of people by age and sex (in quinary bands) [32], and the latest modelled estimates of ethnicity in 2008 and 2009 derived from self-reported, principally closed option, responses in the 2001 national census supplemented by data on births, deaths and migration in the following years [33]. The age and sex data enabled calculation of the percentage of each primary care trust population aged 65 years or more for each time period.

Socio-economic deprivation was expressed using the Index of Multiple Deprivation 2007 (IMD 2007), which, using an agreed formula, combines a set of indicators in 7 domains (income, employment, health, education, housing, crime, and environment) into a single score, which is a relative measure specific for IMD, for each small area in England [34]. The health domain is weighted at 13.5% of the total IMD score, but contains only 1 direct measure of mortality. Although removing the health domain “represents best practice”, it would be likely to have “little practical effect on measured socioeconomic inequalities” [35] in our measures of mortality and a re-calculation would be difficult to undertake satisfactorily at trust population level. Currently, IMD scores are generally accepted as the standard measure of deprivation in England. IMD scores at primary care trust level were obtained from the Department for Communities and Local Government [36].

**Table 5:** Interpretation of associations between primary care predictors and mortality rates for combined model using a generalized estimating equations approach.

Mortality group	Predictor	Interpretation of IRR
CHD	% GP registered list on hypertensionregister	After adjusting for other predictors, the CHD mortality rate was predicted to be 22% lower in the PCT with the highest % of this predictor than in the PCT with the lowest % of this predictor.
Stroke	% GP registered list on hypertensionregister	After adjusting for other predictors, the stroke mortality rate was predicted to be 39% lower in the PCT with the highest % of this predictor than in the PCT with the lowest % of this predictor.
Cancer	% patients with recalled perceptionof being able to see preferred GP	After adjusting for other predictors, the cancer mortality rate was predicted to be 1.2% lower in the PCT with the highest % of this predictor than in the PCT with the lowest % of this predictor.
COPD	% patients with recalled perceptionof being able to see preferred GP	After adjusting for other predictors, the COPD mortality rate was predicted to be 2.8% lower in the PCT with the highest % of this predictor than in the PCT with the lowest % of this predictor.

Obesity and smoking rates in adults were obtained from the Network of Public Health Observatories using the most recent data available, modelled for the 3 year period 2006 to 2008 [37]. Also obtained were the Quality and Outcomes Framework registers for diabetes maintained by general practices for the two corresponding business years 2008/9 [38] and 2009/10 [39], the year running from April 1^st^ to March 31^st^. General practice registers have become a more reliable indicator of diabetes prevalence [40].

#### Service predictors

Selected Quality and Outcomes Framework data were obtained for the two corresponding business years 2008/09 [41] and 2009/10 [42] (where available) for all 152 primary care trusts, in which a total of 8,229 practices reported in 2008/09, and 8,305 reported in 2009/10. The Quality and Outcomes Framework includes indicators relating to the care of several conditions, and the achievement of specified levels of performance for each indicator contributes to the level of payment awarded [43], [44]. The variables from this and other sources, relating to interventions with potential to influence mortality, were grouped according to our conceptual model into:

- *Prevention*. For all cause, cancer and chronic obstructive pulmonary disease mortality, we used the percentage of those attending National Health Service smoking cessation clinics who self-reported quitting smoking at 4 weeks to calculate smoking cessation rates in 2008/09 [45] and 2009/10 [46] (prevention in Starfield’s mechanisms [15]). For chronic obstructive pulmonary disease mortality we used the percentage of patients with chronic obstructive pulmonary disease immunised against influenza (Quality and Outcomes Framework) [41], [42]. For all-cause mortality we used rates of influenza immunisations in those aged 65 years or more in 2008/09 [47] & 2009/10 [48].
- *Early detection*. For all cause, coronary heart disease and stroke mortality, we used the Quality and Outcomes Framework hypertension registers in 2008/09 [38] and 2009/10 [39]. Since only about half of the people in England with hypertension are detected [49], these registers should be regarded as measures of detection, rather than of prevalence, in contrast to diabetes registers.
- *Disease management*. For coronary heart disease mortality, we used the percentage of coronary heart disease patients with a record in the previous 15 months that aspirin, an alternative anti-platelet therapy, or an anti-coagulant was being taken (unless a contraindication or side-effects are recorded) (Quality and Outcomes Framework indicator) and the percentage of coronary heart disease patients whose last measured total cholesterol (measured in the previous 15 months) was 5 mmol/l or less (Quality and Outcomes Framework indicator) [42], [43]. For stroke mortality, we used the percentage of stroke patients with a record in the previous 15 months that aspirin, an alternative anti-platelet therapy, or an anti-coagulant was being taken (unless a contraindication or side-effects are recorded) (Quality and Outcomes Framework indicator), and the percentage of stroke patients whose last measured total cholesterol (measured in the previous 15 months) was 5 mmol/l or less (Quality and Outcomes Framework indicator) [41], [42].
- *Access and sustained relationship*. From the annual General Practice Patient Survey for the years 2008/09 and 2009/10, we used the question on being able to consult a preferred general practitioner, an indication not only of the ability to access care, but also whether patients who so prefer do have access to a sustained relationship, reflecting continuity and the cumulative features of primary care in Starfield’s framework. The patient survey was administered to samples of adults aged 18 years or over in each practice in England, and registered with their general practice for at least 6 months. The questionnaire was sent to 5.7 million people in 2008/09 and a similar number in 2009/10 [50]. The survey in 2008/09 was administered in January 2009 [51], and that in 2009/10 was administered in mailings to a quarter of the randomly selected sample in each calendar quarter [52]. For all respondents, patients who expressed a preference for seeing a particular doctor at their practice were asked: “How often do you see the doctor you prefer to see?” with 4 response options: “always or almost always”, “a lot of the time”, “some of the time” or “never or almost never”. The response options, “always or almost always” and “a lot of the time” were defined as positive, indicating better access to continuity, while the other two options were combined, and defined as indicating worse access to continuity.

### Statistical Analysis

Descriptive statistics for mortality rates and candidate predictors were carried out for each year separately. In order to test the study hypothesis we undertook a separate regression analysis for each category of mortality. The data were over-dispersed counts, so an appropriate analysis method was negative binomial regression, using the log of the primary care trust size as an offset to adjust for the fact that trust size, and thus the number at risk, varied between trusts. Analyses were undertaken using SAS version 9.1 [53]. Sample size was dictated by the total number of primary care trusts in England. We tested the association between mortality counts and primary care characteristics by using a generalized estimating equations approach in which the two years were modelled jointly as a panel model [25].

Population level predictors assumed to have a major impact on mortality, after adjusting for other predictors, were: deprivation; the percentage of the population aged 65 years or over; diabetes registers; the percentage of the population with white ethnicity; the percentage of male patients.

The service variables related to the conceptual model were entered into the regression analysis along with all the population level predictors. The deprivation score and the percentages of adults who were obese and who were smokers were the same variable in each year; otherwise all variables were specific to each year. The significance level (alpha) was set at 0.05.

Interactions between time period (year) and any significant primary care variables were tested to see whether or not effects on the outcome are similar in the two years.

### Role of the Funding Source

The funding organisations had no role in the design and conduct of the study; collection, management, analysis, and interpretation of data; and preparation, review, or approval of the manuscript.

## Results

Data were available for all 152 English primary care trusts in both years. Mortality counts and rates for each disease category are shown in Table 1, and the population and service variables are shown in Table 2.

### Negative Binomial Regression

The estimates, 95% confidence intervals and p-values for each explanatory variable in the analyses for the 5 mortality groups are presented in Table 3. There were no significant interactions between the year in which the data were from and any of the significant primary care effects. Table 4 presents the effect on variations in mortality (in each mortality group) for a unit change in significant predictor variables.

#### Population characteristics

The main population predictors of variation in each mortality group were age (percentage aged 65 years or more), deprivation and white ethnicity. The percentage of smokers was a predictor for variations in stroke mortality. The Quality and Outcomes Framework diabetes register was a predictor for variations in all cause, coronary heart disease and stroke mortality. The modelled percentage of obese adults was a predictor for stroke mortality and for all cancer mortality. The percentage of males was a predictor for chronic obstructive pulmonary disease mortality.

#### Service characteristics

Only two primary healthcare variables were predictors of variations in a mortality group.

Higher levels of detected hypertension (hypertension registers) were significantly associated with lower levels of coronary heart disease and stroke mortality. Being better able to see a preferred doctor was significantly associated with lower levels of chronic obstructive pulmonary disease and cancer mortality.

An effect for time period (year) was included in each model, together with its interaction with the significant primary care predictor, in order to determine whether the primary care effect differed from year to year, but the interaction between time and each significant primary care variable was not significant in any of the models. To better understand the quantitative effect of these primary healthcare predictors, table 5 presents the predicted difference in mortality rates between the PCTs with the highest and lowest percentage for each significant predictor, after adjusting for other predictors.

We found no association between any of the other healthcare variables and variations in trust population mortality in any of the disease groups in our models.

The analyses were repeated using two potential confounding explanatory variables, rates of exception-reporting for the Quality and Outcomes Framework and the percentage of single-handed general practices, which had not been included in the conceptual model. Neither of these variables was a predictor of variations in mortality in any of the analyses. We used the percentage of the population aged 65 years and over as a variable to account for the effect of population on mortality, but other measures of population age might have been selected, and on checking our age correction factor, a correlation of 0.99 was identified between the percentages of the population aged 65 years or more and those aged 75 years or more in each year.

## Discussion

### Main Findings

As expected, variations in mortality rates between primary care trust populations for all-cause mortality and the four specific disease mortality groups were predicted mainly by variations in population factors, particularly deprivation. However, the stated aim of the study was to examine the association between primary healthcare service factors and mortality, with population factors included in the model solely in order to adjust for their effects.

With respect to demographic factors: indices of deprivation are composites of both health and non-health factors. The strongly positive predictive effect of deprivation is expected and consistent with previous work [4], and we note, but cannot explain, the independent positive predictive effect of white ethnicity, an unexpected but consistent finding across all of the mortality groups examined.

With respect to primary healthcare service characteristics, the overall null hypothesis was rejected in each of the four specific disease mortality groups: variations in primary healthcare service factors predicted variations in mortality at the population level, after adjusting for population characteristics. The study’s inability to find a health care predictor for variations in all-cause mortality is perhaps not surprising, due to the heterogeneity of causes of mortality. The negative association of variations in hypertension registers with variations in coronary heart disease and stroke mortality can be explained by the under-detection of hypertension. Thus, the findings suggest that improving detection of hypertension has substantial potential to reduce coronary heart disease and stroke mortality. The much smaller, but still significant, predictive effect of being able to see a preferred doctor (linked to both access and sustained partnership) on variations in cancer and chronic obstructive pulmonary disease mortality may be explained by the mechanisms identified by Starfield, including continuity, comprehensiveness and coordination [15] (better partnerships with individual patients are more likely to expose a greater percentage of the population to appropriate interventions). We found a strong negative correlation between deprivation and patients’ perception of being able to see a preferred doctor (−0.53), suggesting that barriers to forming sustained partnerships with healthcare are greater in deprived populations.

Although the predictive effect of primary care characteristics on variations in mortality is smaller than that of population characteristics, small improvements to primary care performance, when applied to large populations, could produce numerically large benefits, as suggested by tables 4 and 5.

The finding that variations in clinical performance (for example, achieving cholesterol targets in known patients with coronary heart disease or stroke) at population levels (i.e. primary care trust), did not have predictive ability does not mean that clinical management is unimportant in reducing mortality risk in individuals [54]; but rather, the extent of variation was not sufficient to explain variations in mortality in our model at primary care trust level, and may reflect the Rose perspective on preventive health of small shifts in variables applied to large populations [55].

### Limitations of this Study

The service factors included were limited to those for which good quality data were available. Variations in other primary care characteristics, secondary healthcare delivery and non-health care factors may possibly have predictive ability for variations in population mortality.

As discussed in the methods section above, the Index of Multiple Deprivation Score has a health domain weighted at 13.5%, including a single mortality indicator (years of potential life lost). The extent to which any correlation between our mortality dependent variables and the overall IMD score would be exaggerated by the presence of the years of life lost indicator is likely to be small [56]. However, the advantages of using the generally recognized indicator, underpinned by robust methodology, and the lack of a superior alternative, outweighed this small disadvantage.

The data were not entirely contemporaneous; smoking and obesity rates were from 2006–2008; the deprivation index was calculated for 2007; mortality data were for the calendar year; and the Quality and Outcomes Framework, smoking cessation and General Practice Patient Survey data relate to the business year (April to March). These were the latest or best contemporaneous fit for the variables analyzed.

Because of relatively low response rates in the General Practice Patient Survey (22 to 55%), findings using these data have to be interpreted with caution. However, by following rigorous probability sampling processes, these surveys have minimised bias due to low response rates [57]. Also, the surveys were the only source of uniform data on patients’ ability to consult a preferred doctor.

The study was limited to investigating associations at primary care trust level. However, since the basis of the study was a conceptual model with a framework explaining the modifying effect of primary care on population health outcomes [15], and the findings were consistent with the model, the identified associations almost certainly reflect how primary care features affected population mortality.

### Implications

Healthcare reforms should aim to reduce variations in population mortality. This study’s findings indicate that primary care’s effectiveness will be improved when primary care organisations have a strong public health perspective. In such an approach, strategies and incentives should deliver appropriate interventions, including prevention and early detection, to as many people with potential to benefit as possible, in the context of sustained partnership [58] and be tailored to address local needs and the adverse effects of social factors on health [4].

### Conclusions

Primary healthcare service characteristics are associated with variations in mortality rates in England, after adjustment for population characteristics. To reduce these variations, the consistent delivery of simple, cost-effective and appropriate interventions to whole populations is needed, fostered through sustained relationships between patients and healthcare.

#### Ethical committee approval

Not required, as the study used only data that were already collected and publically available, with no individuals or practices identified.
